# Physician Empathy Interacts with Breaking Bad News in Predicting Lung Cancer and Pleural Mesothelioma Patient Survival: Timing May Be Crucial

**DOI:** 10.3390/jcm7100364

**Published:** 2018-10-17

**Authors:** Sophie Lelorain, Alexis Cortot, Véronique Christophe, Claire Pinçon, Yori Gidron

**Affiliations:** 1 University of Lille, CNRS, CHU Lille, UMR 9193—SCALab—Cognitive and Affective Sciences, F-59000 Lille, France; veronique.christophe@univ-lille.fr (V.C.); yori.gidron@univ-lille.fr (Y.G.); 2 University of Lille, Department of Thoracic Oncology, Albert Calmette University Hospital, F-59000 Lille, France; alexis.cortot@chru-lille.fr; 3 University of Lille, CHU Lille, EA 2694, Public Health: Epidemiology and Quality of Care, F-59000 Lille, France; claire.pincon@univ-lille.fr

**Keywords:** empathy, physician—patient relations, truth disclosure, oncology service, hospital, outpatients, lung neoplasms, survival analysis, proportional hazards models, psychology, medical

## Abstract

This study is the first to examine the prognostic role of physician empathy in interaction with the type of consultation (TC) (TC, bad news versus follow-up consultations) in cancer patient survival. Between January 2015 and March 2016, 179 outpatients with thoracic cancer and a Karnofsky performance status ≥60 assessed their oncologist’s empathy using the CARE questionnaire, which provides a general score and two sub-dimensions: listening/compassion and active/positive empathy. Survival was recorded until April 2018. Usual medical, social and psychological confounders were included in the Cox regression. The median follow-up time was 3.1 years. There was a statistical interaction between listening/compassion empathy and TC (*p* = 0.016) such that in bad news consultations, higher listening/compassion predicted a higher risk of death (hazard ratio (HR) = 1.13; 95% confidence interval (CI): 1.03–1.23; *p* = 0.008). In follow-up consultations, listening/compassion did not predict survival (HR = 0.94; 95% CI: 0.85–1.05; *p* = 0.30). The same results were found with the general score of empathy, but not with active/positive empathy. In bad news consultations, high patient-perceived physician compassion could worry patients by conveying the idea that there is no longer any hope, which could hasten death. Further studies are warranted to confirm these results and find out the determinants of patient perception of physician empathy.

## 1. Introduction

Lung cancer is one of the most fatal types of cancer, with 30% survival at one year post-diagnosis of non-small cell lung cancer [1]. One way to improve the prognosis is to identify modifiable prognostic factors such as physical exercise, diet, or patient care. A major meta-analysis showed that psychological factors such as depression or coping predicted prognosis in cancer [2]. However, physician empathy, i.e., the physician’s ability to understand the affective and physical experiences of patients and convey this understanding to them [3], was not part of this meta-analysis. Yet, in other conditions, physician empathy has been associated with multiple health outcomes such as HbA1C or cholesterol in diabetic patients for example [4].

In oncology, associations between physician empathy and various patient outcomes have been shown [5,6]. However, the role of patient perception of physician empathy in cancer survival has never been examined. The main objective of this study was thus to study the prognostic role of patient perception of physician empathy in cancer patient survival. Related to empathy, the role of social support in survival, including cancer survival, has been established [7,8]. As a major source of support for cancer patients, physician empathy may impact survival [9], perhaps especially in the context of informing patients on their disease progression. In such a personally meaningful context of receiving bad news, it is plausible that most patients attend carefully to the content and manner of the physician’s speech. How physicians present the progress of the disease and its treatment to patients could play a major role in counteracting the potential helplessness/hopelessness of patients [10], which are associated with poorer prognosis [11,12]. Biologically, empathy is also related to the hormone oxytocin [13], which has anti-proliferative, anti-metastatic and anti-angiogenic effects in some cancers [14].

Furthermore, the effect of empathy on prognosis could vary according to the type of consultation (TC). For example, in an emotionally ‘neutral’ consultation (i.e., follow-up of treatment), physician empathy may have a weak effect on patients since patients are theoretically not distressed. Conversely, when bad news is being given, the effects of empathy could be stronger due to greater patient distress [15,16]. Thus, we hypothesized that higher physician empathy would be related to a lower risk of death in bad news consultations but not in follow-up consultations (interaction hypothesis).

Finally, the definition of medical empathy is far from consensual [17]. However, two types of empathy can be distinguished: a rather passive empathy of listening and compassion, whereby the physician listens attentively to patients and shows them compassion; and an active and positive empathy whereby the physician tries to give control and options to patients (e.g., providing a great deal of information and shared-decision making) and stays positive [18]. We thus tested whether the hypothesized interaction between empathy and type of consultation, in relation to survival, was valid for both types of empathy or not (secondary objective). Multiple sociodemographic and medical confounders were taken into account. Two psychological confounders—patient cancer-related distress and patient emotional skills (i.e., patient skills in identifying, understanding, expressing and regulating emotions)—were also considered for their known role in cancer progression [2,19,20,21,22,23], as they could modify the influence of empathy on survival.

## 2. Experimental Section

### 2.1. Participants

Inclusion criteria for physicians were treating thoracic cancer patients in an outpatient hospital setting. Inclusion criteria for patients were thoracic cancer at all stages or types of cancer, outpatients ≥18 years old, aware of the cancer diagnosis and with a Karnofsky Performance Status (KPS) ≥60. Performance status is a score that estimates the patient’s ability to perform certain activities of daily living without the help of others. These daily activities include basic activities such as getting dressed, eating, and bathing, as well as more complex activities such as cleaning the house and working a regular job [24]. The scores range from 0 “dead” to 100 “normal”. For example, a patient at 60 requires occasional assistance and cares for most needs. Exclusion criteria were patients for whom there was no clear information regarding disease progression (e.g., waiting for test results) and those with a psychiatric disorder.

### 2.2. Design and Procedure

The data used were from an earlier study whose results had been reported previously [25]. The study was approved by the French national ethics committee (approval number 14.545). In short, during the initial study, physicians working in the thoracic cancer department of the University of Lille (France) proposed the study to patients meeting the inclusion criteria at the end of a consultation. After the consultation, interested patients signed an informed consent and completed a questionnaire assessing their physician’s empathy, their own cancer-related distress, emotional skills and socio-demographic data (see below for details). The type of consultation was reported by the physicians just after the consultations. Medical and clinical data—Karnofsky index, cancer stage, type of cancer, type of treatments before the study, Charlson index of comorbidities, metastases, mutations epidermal growth factor receptor (EGFR), anaplastic lymphoma kinase (ALK) or ROS proto-oncogene 1, receptor tyrosine kinase (ROS), smoking status and time since diagnosis to study entry–were retrieved by the research assistants. The initial study ran from January 2015 to July 2016. For the present extended study, patients were followed until 1 April 2018, the date of censoring. At this point, research assistants added the survival status and treatments received by patients after the initial study.

### 2.3. Measures

Patient perception of physician empathy was measured using the Consultation and Relational Empathy (CARE) measure, a 10-item 5-point Likert scale [26]. Items of this scale deal with the patient’s perception of physician listening, respect, clear explanations and information provision, whether the physician fully understood his/her concerns, showed care and compassion, tried to be positive while staying honest, helped him/her to take control, and made an action plan with him/her. While the scale was designed to provide a global score, studies haves suggested that it could be divided into two subscales [18,26,27]: a listening/compassion score (items 1 to 6) and an active/positive empathy score (items 7 to 10). Cronbach’s α was 0.95 in our sample for the global score, 0.93 for the listening/compassion score, and 0.91 for the active/positive empathy score.

Cancer-related distress was assessed using the emotional dimension of the Functional Assessment of Cancer Therapy-General (FACT-G), a 6-item 5-point Likert scale [28]. Higher scores represent higher distress. Examples of items are “I feel sad”, “nervous”, “I worry about dying”, and “I am losing hope in the fight against my illness”.

Patient emotional skills were assessed using the Short-Profile of Emotional Competence (S-PEC) scale [29], a 20-item 5-point Likert scale providing a score of emotional skills (e.g., how patients identify, understand, express and regulate their emotions and the emotions of others in general). Examples of items are: “When I feel good, I can easily tell whether it is due to being proud of myself, happy or relaxed”, “It is easy for me to explain my feelings to others” or “I find it difficult to handle my emotions” (reversed). Higher scores represent higher emotional skills.

The type of consultation was reported by the physician at the end of the consultation: if the patient was informed of a cancer progression in spite of treatment, or cancer relapse or the end of active treatment, this was considered a “bad news consultation”. Otherwise, it was a “follow-up consultation”.

Sociodemographic data were self-reported by patients and included age, gender, education and financial status.

Medical data were obtained from medical charts and included the Karnofsky index, cancer stage, type of cancer, Charlson index of comorbidities, metastases, mutation types (AGFR, ALK or ROS), treatments before and after the study entry, and time between diagnosis and study entry.

### 2.4. Statistical Analyses

Continuous variables are expressed as mean ± SD. Categorical variables are presented as absolute numbers and percentages. Distributions were examined for outliers. For categorical variables, frequencies were inspected and categories were merged when there were insufficient cases in one or more categories. Median follow-up was estimated using the inverse Kaplan-Meier method. Whether covariates differed between bad news and follow-up consultations was examined using independent sample *t* tests or Pearson χ². The univariate association of survival with each potential covariate was assessed using Cox proportional hazard regression models. From the fitted Cox models, the unadjusted hazard ratio with 95% CIs was obtained, and the significance of the association was tested using the Wald χ² statistic. Log-linearity and proportional hazards assumptions were checked for each covariate using Martingale and Schoenfeld residuals as well as supremum tests. If the proportional hazards assumption was not met, a piecewise model was created. The covariates that were tested included gender, age, education (no diploma, high school, bachelor degree vs. > bachelor degree), financial situation (not comfortable, moderately comfortable vs. very comfortable), type and severity of cancer (Non-Small Cell Lung cancer (NSCL)-Small Cell Lung cancer (SCL) Stage I, II, III vs. NSCL-SCL stage IV, mesothelioma), metastases, Karnofsky index of 60–70 vs. >70, time between diagnosis and inclusion in the study in years, Charlson index, mutation EGFR or ALK or ROS vs. none of these, cancer-related distress and patient emotional skills. All covariates except smoking status (too many missing data) and treatments (many treatments and high correlations with other medical data) were included in the multivariate model. Although no prior sample size calculation was done for this data analysis because of the retrospective design of this analysis, attention was paid to roughly respect the rule of 10 events per covariate [30,31] in spite of debates about this rule of thumb [32,33]. To choose between two non-nested models, the Schwarz Bayesian Criterion (SBC) was used, a smaller SBC indicating a better fit (at least two points of differences are required). To illustrate the effect of empathy on survival by the type of consultation with Kaplan-Meier curves, empathy was dichotomized at the median of 45. Curves were plotted, controlling for confounders, with continuous variables set at their means and categorical variables set at their mode. A two-tailed type I error rate <0.05 was considered for statistical significance. Analyses and graphs were performed using SAS version 9.4 (SAS Institute Inc., Cary, NC, USA).

## 3. Results

### 3.1. Descriptive Statistics

Among the 179 patients in the total sample, the median follow-up was estimated at 3.1 years, 95% CI (2.92–3.09). Eighty-three patients (46.4%) were still alive at the time of censoring, 1 April, 2018 (Table 1). Patients were mostly older men with a relatively low level of education, non-small cell lung cancers at stages I, II or III, a Karnofsky score indicating correct functional status. Forty three percent of patients were included in the study after the disclosure of bad news, consisting overwhelmingly of a change of treatment due to treatment failure. Five physicians participated in the study to present it to eligible patients and collect their agreements.

Table A1 (Appendix A) shows the comparisons of the “bad news consultation” sample to the “follow-up consultation” one for all socio-demographic variables. Expected differences were observed between the two samples regarding the type and stages of cancer, the presence of metastases, the treatments received, the comorbidity index and the number of deaths at study censoring. At the psychological level, perceived empathy did not differ between bad news consultations and follow-up ones. Patients were also similar in emotional skills. As expected, however, cancer-related distress was higher after a bad news consultation than after a follow-up one.

Table A2 (Appendix A) shows the comparisons of the low versus high patient-perceived physician empathy groups. The two groups were identical with the exception of three variables: there was a higher proportion of men in the low patient-reported empathy group compared to the other group (high empathy), cancer-related emotional distress was higher in the low empathy group, and patients had a lower level of emotional skills in the low empathy group.

### 3.2. Prediction of Survival with the General Score of Empathy

The univariate analyses (Table 2) showed that age, comorbidities, cancer-related distress, stage IV cancers and mesotheliomas (compared to stages I, II, or III), metastasis and receiving chemotherapy before and/or after inclusion in the study were associated with an increased risk of death. Conversely, being a woman, having had surgery before the study, and being without additional treatment after inclusion in the study were protective factors for survival.

**Figure 1:**
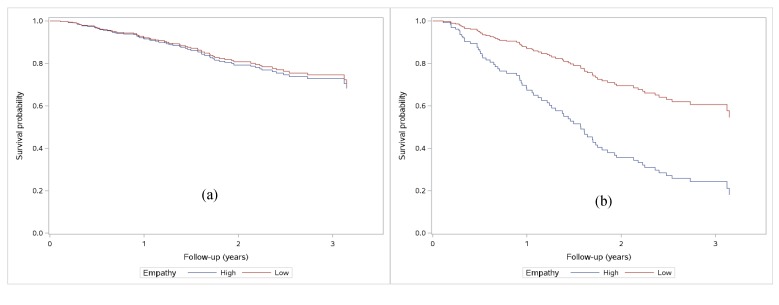
Survival probability for patients with high perceived empathy (>45) versus low empathy (≤ 45) by median split. (**a**) Follow-up consultations: adjusted Cox regressions showed no differences by empathy (*p* = 0.24); (**b**) Bad-news consultations: adjusted Cox regressions showed differences by empathy (*p* = 0.024).

In the multivariate analysis (Table 3), age, stage IV or mesothelioma, and cancer-related distress increased the risk of death. On the contrary, being a woman and having a high school diploma (compared to no diploma) were protective factors for survival. Controlling for sociodemographic and medical variables, the interaction between the type of consultation and patient-perceived empathy was significant (*p* = 0.02). However, it went in the opposite direction to our hypothesis. While, as hypothesized, empathy was not related to survival in follow-up consultation, HR = 0.96 by point of empathy score, 95% CI (0.90–1.03), it unexpectedly increased the risk of death by 6%, 95% CI (1.01–1.12) in bad news consultations (Figure 1). Thus, a one-point increase in the empathy questionnaire increased the risk of death by 6% in patients receiving bad news.

To be sure that treatments did not change the results, in an alternative model, we introduced treatments associated with survival at *p* < 0.05 in the univariate analyses. This did not change the results, but the fit of this alternative model (SBC = 689.8) was not as good as that of the model presented in Table 3 (SBC = 668.8) and therefore it was not retained.

### 3.3. Prediction of Survival by Distinguishing the Two Types of Empathy

In order to determine if the interaction was due to the two types of empathy, the model was reiterated identically but general empathy was first replaced by listening and compassion empathy (model A) and then by active and positive empathy (model B). The results are presented in Table 4.

A significant interaction was found only with the empathy of listening and compassion. This type of empathy was associated with an increased risk of death in the bad news condition, HR = 1.13 by point of score, 95% CI (1.03–1.23), *p* = 0.008, but not in the follow-up condition, HR = 0.94 by point of score, 95% CI (0.86–1.05), *p* = 0.30. In contrast, active and positive empathy did not interact with the type of consultation, (*p* = 0.07). Regardless of the type of consultation, active/positive empathy was not associated with survival. Interestingly, both models detailing a specific type of empathy had a better fit (SBC of 556.3 for listening/compassion and 649.5 for active/positive empathy) than the model with general empathy (SBC of 668.8).

## 4. Discussion

To our knowledge, these are the first data examining the relationship between patients’ perceived oncologist empathy and survival in lung cancer. Unexpectedly, in the bad news consultations, patient-perceived empathy was associated with a higher risk of death after controlling for sociodemographic, psychological and biomedical variables. It should be remembered that in our sample, mean perceived-empathy was high compared to other samples [34]. In fact, a high level of physician empathy can worry patients and convey the message that the situation is really bad [35], which could hasten death. Indeed, previous studies have shown that patient awareness of the palliative nature of the situation negatively impacts their survival [36,37,38,39]. The fact that only compassion and listening empathy was associated with death is consistent with the hypothesis that highly unusual compassion and listening from physicians could be harmful, as it may equate to a loss of hope in patients’ mind. On the contrary, positive and active empathy, which is by definition less worrying and more oriented towards an action plan, was not linked to survival status in our sample. Delivering bad news honestly while remaining positive and focused on an action plan is probably thus the challenge for physicians in order to maintain patient hope and control, as written in the American Society of Clinical Oncology guideline [40]. The challenge is the same for patients: to maintain a feeling of hope and control in spite of bad news.

This study had limitations. First, the mean empathy was high so that our results need to be replicated in samples with lower empathy. Second, the generalizability of findings to other samples with different characteristics needs to be tested. In particular, our sample was mostly composed of male patients and the results may be totally different for female patients suffering from other cancers. Indeed, female cancer patients are much more sensitive to physician attitude, support, communication and information than male patients [41]. Besides, in our sample, being a female patient was a protective factor. It might be the case because women are generally more prone to search and find social support, which is positively associated with higher survival [8]. Our sample was also composed of a majority of people with a low level of education. High school diploma was a protective factor compared to patients with no diploma, probably as it allows a feeling of control and a better health literacy. A sample with a higher education level could have been less sensitive to physician empathy. Indeed, cancer patients with a high education are less sensitive to physician communication and attitude and rely more on alternate sources of information than patients with a low education level [41,42]. The results could also be the opposite (as initially hypothesized) for cancers with a better prognosis. Finally, the results are limited by the exploratory nature of these post-hoc analyses and by the patient-reported measure of physician empathy. Further studies could consider more survival predictors such as immunohistochemistry biomarkers (e.g., p16 or BAP1 in pleural mesothelioma [43]), risk profiles (i.e., a combination of risk factors, which predicts survival [44]), or more specific treatments (e.g., PD-1 PD-L1). Taking into account these limitations, further evidence is essential to provide more definitive conclusions.

## 5. Conclusions

In bad news consultations, higher patient perception of physician listening and compassion empathy was associated with a higher risk of death in lung cancer patients. These results could have serious implications for physician-patient communication training as, if they were further confirmed, they would challenge the pervasive and implicit idea that physicians must always be compassionate and would call for a clarification of what is meant by ‘empathy’. Research demonstrating weak or mixed evidence of the effectiveness of recommended guidelines for breaking bad news such as the six-step SPIKES protocol [42,45,46], which promotes an empathic attitude, suggest that such “breaking-bad-news rules” do not meet all patients’ needs [47]. In fact, evidence-based research is really warranted regarding breaking bad news. Further research would then be necessary to elucidate what physician behaviors or words are related to the patient-perception of physician empathy, which in turn is related to better patient outcomes. Further research would also be needed to determine what patient factors (e.g., personality, medical history) might explain the patient-perception of physician empathy and patient outcomes.

## Figures and Tables

**Table 1:** Descriptive statistics.

Characteristics	Number of Patients	%	Mean (Standard Deviation) (Range)
Patients (*n* = 179)			
Sociodemographic data			
Age			63.4 (11.3) (24–85)
Men	121	67.6	
In a relationship or married (Yes)	135	76.2	
Patient-reported education			
No diploma	39	21.8	
High school	90	50.3	
Bachelor degree	33	18.4	
More than bachelor degree	17	9.5	
Patient-reported financial situation			
Not at all or not very comfortable	28	15.6	
Moderately comfortable	100	55.9	
Rather or very comfortable	51	28.5	
Medical data			
Type of cancer			
Non-small cell lung cancer (NSCL)	135	75.4	
Small cell lung cancer (SCL)	10	5.6	
Mesothelioma	21	11.7	
Missing data	13	7.3	
Cancer stage			
Stage I, II or III (NSCL or SCL)	84	46.9	
Stage IV (NSCL or SCL)	56	31.3	
Mesothelioma (no stage assigned)	21	11.7	
Missing data	18	10.1	
Smoker			
Never	24	13.4	
Ever	106	59.2	
Missing data	49	27.4	
Time since diagnosis and inclusion in the study (years)			2.5 (2.2) (0.1–14.4)
Karnofsky index			
60–70	8	4.5	
80	20	11.2	
90	56	31.2	
100	89	19.7	
Missing data	6	3.3	
Metastasis	51	28.5	
Treatment before inclusion			
Surgery	82	45.9	
Chemotherapy	119	66.5	
Radiotherapy	71	39.7	
Targeted therapies	14	7.8	
Treatment after inclusion			
Surgery	8	4.5	
Chemotherapy	80	44.7	
Radiotherapy	38	21.2	
Targeted therapies	31	17.3	
Immunotherapy	36	20.1	
No further treatment	60	33.5	
Charlson index of comorbidities			8.0 (2.6) (0–16)
Genetic mutation: EGFR or ALK or ROS (%)	20	11.2	
Type of consultation			
Routine follow-up	100	55.9	
Bad news	79	43.0	
Change of treatment because of treatment failure %	69	38.6	
Relapse or end of active treatment	8	4.5	
Missing data	2	1.1	
Deceased at censorship			
Yes	88	49.2	
No	83	46.4	
Missing data	8	4.4	
Psychological data			
Cancer-related emotional distress			8.5 (5.2) (0–24)
Patient emotional skills			3.4 (0.5) (1.9–4.8)
Patient-reported physician empathy			43.2 (6.8) (22–50)
Physicians (*n* = 5)			
Age			35.8 (5.85) (33–48)
Men		50	

NSCL: Non-small cell lung cancer; SCL: Small cell lung cancer; EGFR: Epidermal Growth Factor Receptor; ALK: Anaplastic Lymphoma Kinase; ROS: Proto-oncogene tyrosine-protein kinase. ROS is an enzyme that in humans is encoded by the *ROS1* gene.

**Table 2:** Unadjusted Cox Proportional Hazard Ratios for overall survival.

Variable	HR	95% CI	*p* Value
Sociodemographic data			
Age	1.02	1.01–1.04	0.015
Woman	0.51	0.30–0.84	0.009
In a relationship or married (Yes)	0.88	0.52–1.47	0.62
Patient-reported Education			
Overall			0.49
No diploma	1.97	0.79–4.92	0.15
High school	1.47	0.63–3.46	0.37
Bachelor degree	1.58	0.62–4.00	0.34
More than bachelor degree (reference category)			
Patient-reported financial situation %			
Overall			0.25
Not at all or not very comfortable	0.55	0.27–1.12	0.10
Moderately comfortable	0.84	0.53–1.32	0.44
Rather or very comfortable (reference category)			
Medical data			
Type and severity of cancer			<0.0001
Stage I, II or III (NSCL or SCL) (reference category)			
Stage IV (NSCL or SCL)	2.48	1.51–4.05	0.0003
Mesothelioma (no stage assigned)	3.36	1.81–6.23	0.0001
Smoker (ever)	0.89	0.45–1.78	0.75
Time since diagnosis and inclusion in the study (years)	0.96	0.87–1.07	0.46
Karnofsky index (60–70 compared to higher)	2.11	0.92–4.85	0.08
Metastasis %	1.63	1.04–2.56	0.03
Treatment before inclusion %			
Chemotherapy	1.78	1.06–2.90	0.02
Surgery	0.34	0.21–0.54	<0.0001
Radiotherapy	0.95	0.62–1.45	0.80
Targeted therapies	1.83	0.92–3.64	0.09
Treatment after inclusion %			
Surgery	0.17	0.02–1.25	0.08
Chemotherapy	2.18	1.41–3.37	0.0005
Radiotherapy	0.65	0.38–1.13	0.13
Targeted therapies	1.56	0.95–2.54	0.08
Immunotherapy	1.59	0.99–2.55	0.06
No further treatment	0.48	0.29–0.81	0.006
Charlson index of comorbidities	1.19	1.09–1.31	<0.0001
Genetic mutation: EGFR or ALK or ROS (%)	1.22	0.67–2.25	0.52
Psychological data			
Cancer-related emotional distress	1.05	1.01–1.09	0.01
Patient emotional skills	0.87	0.59–1.29	0.48
Patient-reported physician empathy	0.99	0.97–1.03	0.87

NSCL: Non-small cell lung cancer; SCL: Small cell lung cancer; HR: hazard ratio; CI: confidence interval. EGFR: Epidermal Growth Factor Receptor; ALK: Anaplastic Lymphoma Kinase; ROS: ROS proto-oncogene 1, receptor tyrosine kinase.

**Table 3:** Adjusted Cox Proportional Hazard Model for overall survival.

Variable	Hazard Ratio	95% CI	*p* Value
Age	1.04	1.00–1.07	0.029
Woman	0.48	0.26–0.89	0.019
Patient-reported education			
No diploma (reference)			
High school	0.42	0.22–0.81	0.009
Bachelor degree	0.41	0.16–1.06	0.07
>Bachelor	0.50	0.14–1.70	0.26
Patient-reported financial situation			
Not at all or not very comfortable (reference)			
Moderately comfortable	1.42	0.58–3.45	0.44
Rather or very comfortable	1.27	0.46–3.57	0.65
Type and severity of cancer			
Stage I, II or III (NSCL or SCL) (reference category)			
Stage IV (NSCL or SCL)	3.14	1.22–8.09	0.018
Mesothelioma (no stage assigned)	3.30	1.22–8.91	0.018
Karnofsky index 60–70 (compared to >70)	2.53	0.84–7.59	0.10
Time since diagnosis and inclusion in the study	0.98	0.84–1.14	0.75
Charlson index of comorbidities	1.02	0.88–1.17	0.80
Mutation (ALK, EGFR, ROS vs. none of them)	1.40	0.64–3.06	0.40
Metastases	0.92	0.38–2.25	0.85
Cancer-related emotional distress	1.06	1.01–1.12	0.03
Emotional skills	1.57	0.87–2.85	0.14
Patient-reported physician empathy	-	-	-
Type of consultation (bad news vs. follow-up)	-	-	-
Empathy * type-of-consultation (interaction)			0.022
Empathy in bad news consultations	1.06	1.01–1.12	0.024
Empathy in follow-up consultations	0.96	0.90–1.03	0.24

NSCL: Non-small cell lung cancer; SCL: Small cell lung cancer; *n* = 143 (due to missing data in some variables), -2LL = 587.8, Akaike Information Criterion (AIC) = 625.8, Schwarz Bayesian Criterion (SBC) = 668.8. EGFR: Epidermal Growth Factor Receptor; ALK: Anaplastic Lymphoma Kinase; ROS: ROS proto-oncogene 1, receptor tyrosine kinase.

**Table 4:** Adjusted Cox Proportional Hazard Models for overall survival with specific types of empathy.

Variable	Hazard Ratio	95% CI	*p* Value
Model A			
Compassion/listening empathy * type-of-consultation (interaction)	-	-	0.016
Compassion/listening in bad news consultations	1.13	1.03–1.23	0.008
Compassion/listening in follow-up consultations	0.94	0.85–1.05	0.300
Model B			
Active/positive empathy * type-of-consultation (interaction)	-	-	0.07
Active/positive in bad news consultations	1.10	0.96–1.26	0.16
Active/positive in follow-up consultations	0.92	0.79–1.06	0.25

Covariates are not shown for readability purposes. Model A: -2LL = 575.6, Akaike Information Criterion (AIC) = 613.6, Schwarz Bayesian Criterion (SBC) = 556.3, *n* = 139; Model B: -2LL = 569.1, AIC = 607.1, SBC = 649.5, *n* = 138. CI: confidence interval.

## Appendix A

**Table A1:** Characteristics of patients in follow-up versus bad news consultations.

Characteristics	Follow-Up Consultations			Bad-News Consultations			*p* Value
	Number of Patients	%	Mean (Standard Deviation, SD)	Number of Patients	%	Mean (SD)	
Patients (*n* = 179)							
Sociodemographic data							
Age			63.4 (11.9)			63.4 (10.5)	0.99
Men	67	66.3		54	69.2		0.68
Married or in a relationship (Yes)	82	81.2		53	69.7		0.08
Patient-reported education							0.67
No diploma	22	21.8		17	21.8		
High school	49	48.5		41	52.6		
Bachelor degree	18	17.8		15	19.2		
More than bachelor degree	12	11.9		5	6.4		
Patient-reported financial situation							0.86
Not at all or not very comfortable	17	16.8		11	14.1		
Moderately comfortable	55	54.5		45	57.7		
Rather or very comfortable	29	28.7		22	28.2		
Medical data							
Type and severity of cancer							<0.0001
Stage I, II or III (NSCL or SCL)	62	61.4		22	28.2		
Stage IV (NSCL or SCL)	22	21.8		34	43.6		
Mesothelioma (no stage assigned)	8	7.9		13	16.7		
Missing data	9	8.9		9	11.5		
Smoker							0.12
Never	12	11.9		12	15.4		
Ever	71	70.3		85	44.8		
Missing data	18	17.8		31	39.7		
Time since diagnosis and inclusion in the study (years)			2.5 (2.4)			2.4 (1.9)	0.64
Karnofsky index							0.76
60–70	5	4.9		3	3.9		
>70	54	93.1		71	91.0		
Missing data	2	2.0		4	5.1		
Metastasis	17	16.8		34	43.6		<0.0001
Treatment before inclusion							
Chemotherapy	57	56.4		62	79.5		0.0012
Surgery	60	59.4		22	28.2		<0.0001
Radiotherapy	47	46.5		24	30.8		0.0325
Targeted therapies	5	5.1		9	11.7		0.10
Treatment after inclusion							
Surgery	6	5.9		2	2.6		0.28
Chemotherapy	31	30.7		49	62.8		<0.0001
Radiotherapy	16	15.9		22	28.2		0.04
Targeted therapies	12	11.9		19	24.4		0.03
Immunotherapy	11	10.9		25	32.1		0.0005
No further treatment	49	48.5		11	14.1		<0.0001
Charlson index of comorbidities			7.6 (2.7)			8.5 (2.2)	0.03
Genetic mutation: EGFR or ALK or ROS (%)	8	7.9		12	15.4		0.12
Deceased at censoring	36	35.6		52	66.7		<0.0001
Psychological data							
Cancer-related emotional distress			7.7 (5.5)			9.6 (4.7)	0.02
Patient emotional skills			3.3 (0.6)			3.3 (0.5)	0.66
Patient-reported physician empathy			43.7 (6.7)			42.5 (7.1)	0.23

NSCL: Non-small cell lung cancer; SCL: Small cell lung cancer; EGFR: Epidermal Growth Factor Receptor; ALK: Anaplastic Lymphoma Kinase; ROS: ROS proto-oncogene 1, receptor tyrosine kinase.

**Table A2:** Characteristics of patients with low versus high patient-reported physician empathy (median split at 45).

Characteristics	Low Patient-Reported Physician Empathy (≤45)			High Patient-Reported Physician Empathy (>45)			*p* Value
	Number of Patients	%	Mean (Standard Deviation, SD)	Number of Patients	%	Mean (SD)	
Patients (*n* = 179)							
Sociodemographic data							
Age			64.1 (12.4)			62.9 (10.3)	0.50
Men	67	74.4		53	60.2		0.04
Married or in a relationship (Yes)	64	72.7		70	79.6		0.29
Education							0.43
No diploma	24	26.7		15	17.1		
High school	44	48.9		46	52.3		
Bachelor degree	15	16.7		17	19.3		
More than bachelor degree	7	7.8		10	11.4		
Financial ease							0.09
Not at all or not very comfortable	13	14.4		15	17.1		
Moderately comfortable	57	63.3		42	47.7		
Rather or very comfortable	20	22.2		31	35.2		
Medical data							
Type and severity of cancer							0.71
Stage I, II or III (NSCL or SCL)	44	48.9		40	45.5		
Stage IV (NSCL or SCL)	26	28.9		29	33.0		
Mesothelioma (no stage assigned)	12	13.3		9	10.2		
Missing data	8	8.9		10	11.4		
Smoker							0.83
Never	12	13.3		12	13.6		
Ever	50	55.6		55	62.5		
Missing data	28	31.1		21	23.4		
Time since diagnosis and inclusion in the study (years)			2.3 (1.9)			2.5 (2.3)	0.57
Karnofsky index							0.18
60–70	6	6.7		2	2.3		
>70	83	92.2		81	92.1		
Missing data	1	1.1		5	5.7		
Metastasis	23	26.7		27	31.0		0.53
Treatment before inclusion							
Chemotherapy	57	63.3		61	69.3		0.40
Surgery	39	43.3		43	48.9		0.46
Radiotherapy	36	40.0		34	38.6		0.85
Targeted therapies	7	7.8		7	8.1		0.95
Treatment after inclusion							
Surgery	4	4.4		4	4.5		0.97
Chemotherapy	44	48.9		35	39.8		0.22
Radiotherapy	23	25.6		15	17.5		0.17
Targeted therapies	15	16.7		16	18.2		0.79
Immunotherapy	19	21.1		17	19.3		0.77
No further treatment	27	30.0		33	37.5		0.29
Charlson index of comorbidities			8.2 (2.5)			7.7 (2.6)	0.29
Genetic mutation: EGFR or ALK or ROS (%)	10	11.1		10	11.4		0.12
Deceased at censoring	45	50.6		42	51.9		0.87
Psychological data							
Cancer-related emotional distress			9.7 (5.1)			7.5 (5.1)	0.006
Patient emotional skills			3.2 (0.4)			3.5 (0.6)	0.005
Patient-reported physician empathy			37.1 (5.0)			48.6 (1.7)	0.001

NSCL: Non-small cell lung cancer; SCL: Small cell lung cancer; EGFR: Epidermal Growth Factor Receptor; ALK: Anaplastic Lymphoma Kinase; ROS: ROS proto-oncogene 1, receptor tyrosine kinase.
